# Knowing what and where: TMS evidence for the dual neural basis of geographical knowledge

**DOI:** 10.1016/j.cortex.2015.11.021

**Published:** 2016-02

**Authors:** Paul Hoffman, Sebastian Crutch

**Affiliations:** a Neuroscience and Aphasia Research Unit (NARU), University of Manchester, UK; b Centre for Cognitive Ageing and Cognitive Epidemiology (CCACE), Department of Psychology, University of Edinburgh, UK; c Dementia Research Centre, Department of Neurodegenerative Disease, UCL Institute of Neurology, University College London, London, UK

**Keywords:** Anterior temporal lobe, Inferior parietal sulcus, Semantic cognition, Magnitude

## Abstract

All animals acquire knowledge about the topography of their immediate environment through direct exploration. Uniquely, humans also acquire geographical knowledge indirectly through exposure to maps and verbal information, resulting in a rich database of global geographical knowledge. We used transcranial magnetic stimulation to investigate the structure and neural basis of this critical but poorly understood component of semantic knowledge. Participants completed tests of geographical knowledge that probed either information about spatial locations (e.g., France borders Spain) or non-spatial taxonomic information (e.g., France is a country). TMS applied to the anterior temporal lobe, a region that codes conceptual knowledge for words and objects, had a general disruptive effect on the geographical tasks. In contrast, stimulation of the intraparietal sulcus (IPS), a region involved in the coding of spatial and numerical information, had a highly selective effect on spatial geographical decisions but no effect on taxonomic judgements. Our results establish that geographical concepts lie at the intersection of two distinct neural representation systems, and provide insights into how the interaction of these systems shape our understanding of the world.

## 1 Introduction

There is a long history of studies investigating how humans and other animals learn about the topography of their environments through direct exploration and navigation. Much of this work has focused on the roles of the hippocampus and parahippocampal regions in topographical learning and in the representation of scenes and environments (Burgess et al., 2002, Hafting et al., 2005, Maguire et al., 1998, O'Keefe and Nadel, 1978). In addition to direct experience, however, humans also learn about locations indirectly through exposure to verbal and non-verbal materials (e.g., travel guides and maps). This learning contributes to a rich database of global geographical knowledge, on a much larger scale than could achieved through direct experience alone (Beatty, 1989, Friedman and Dewinstanley, 2006). This information is integral to a range of everyday situations, from planning journeys and holidays to identifying locations described in news reports. In addition to its relevance in everyday life, geographical knowledge is of considerable theoretical interest. Geographical concepts comprise both spatial (e.g., Spain borders Portugal) and non-spatial (Spain is a hot country) elements and thus offer a unique opportunity to investigate the interaction of the brain's semantic and spatial representation systems (Crutch and Warrington, 2003, Crutch and Warrington, 2010). Almost nothing is known, however, about the neural basis of geographical concepts.

In this study, we investigated the roles of the right intraparietal sulcus (IPS) and left anterior temporal lobe (ATL) in the representation of geographical knowledge. The ATL and IPS are major components in two distinct representational systems specialised for different types of knowledge. In recent years, the ATL has emerged as a key site for representation of semantic knowledge for the meanings of words (Binney et al., 2010, Hoffman et al., 2015, Pobric et al., 2007), properties of objects (Pobric et al., 2010a, Vandenberghe et al., 1996) and the identities of people (Drane et al., 2013, Haxby et al., 2000). The critical role of this region is illustrated clearly by the profound deterioration in these forms of knowledge observed in the syndrome of semantic dementia, a neurodegenerative disorder associated with ATL atrophy (Bozeat et al., 2000, Crutch and Warrington, 2006, Hodges and Patterson, 2007). In contrast, IPS is involved in numerical and spatial processing (Dehaene et al., 2003, Hubbard et al., 2005, Mazard et al., 2004) and, in particular, is thought to be the site of the “approximate number system” – a system involved in abstract representation of numerical magnitudes (Feigenson, Dehaene, & Spelke, 2004). It has been proposed that this region uses a common code to represent not only numerical quantities but also magnitudes in sensory domains, including physical size and distance, temporal duration and luminance (Cohen Kadosh et al., 2008, Hubbard et al., 2005, Walsh, 2003).

Typically, the functions of the ATL “semantic” system and the IPS “magnitude” system are highly dissociable. Patients with ATL damage, for example, exhibit preserved understanding of numerical magnitude (Cappelletti et al., 2001, Cappelletti et al., 2005, Crutch and Warrington, 2002, Diesfeldt, 1993, Jefferies et al., 2004) and are able to estimate quantities accurately (Julien, Thompson, Neary, & Snowden, 2010), despite severe deficits in knowledge for objects and words. Conversely, parietal damage is frequently associated with dyscalculia but relative preservation of verbal semantic knowledge (Dehaene and Cohen, 1997, Delazer et al., 2006, Kas et al., 2011). We predicted, however, that both systems would play important roles in the representation of geographical concepts. Much of our knowledge for locations is acquired through exposure to verbal sources of information hence, in common with other forms of verbal semantic knowledge (e.g., Binney et al., 2010), we predicted that the ATL would support this information. However, unlike most other verbal and object concepts, geographical concepts are strongly associated with a particular location in space and with fixed spatial relationships with other known locations. These relationships are integral to our understanding of them. For this reason, we predicted that parietal lobe regions involved in spatial representation would also make an important contribution to the representation of these concepts.

To test these hypotheses, we used repetitive transcranial magnetic stimulation (rTMS) to temporarily disrupt the function of either IPS or ATL in healthy participants. rTMS is commonly used to investigate the functions of specific cortical regions by inducing temporary neural disruption and observing the effects on cognitive processes of interest. This is often referred to as the “virtual lesion” technique (Walsh & Cowey, 2000). Previous rTMS investigations have implicated the ATL in semantic knowledge for words and objects (Pobric et al., 2007, Pobric et al., 2010a) and IPS in the representation of numerical magnitudes (Dormal, Andres, & Pesenti, 2008; Gobel et al., 2001, Kadosh et al., 2007). Here, we investigated how disruption to these two areas affected spatial and non-spatial aspects of geographical knowledge.

## 2 Method

### 2.1 Participants

Eighteen right-handed participants took part (9 female; mean age = 25). All participants grew up and had spent the majority of their lives in the United Kingdom. All participants provided written consent after being screened for adverse effects of TMS. The experiment was approved by the local ethics committee. At the beginning of the study, participants were asked to rate, on a 7-point scale, their level of geographical knowledge of the UK and the rest of the world. The two ratings were averaged to give a measure of perceived geographical ability.

### 2.2 Tasks probing geographical knowledge

**Fig. 1:**
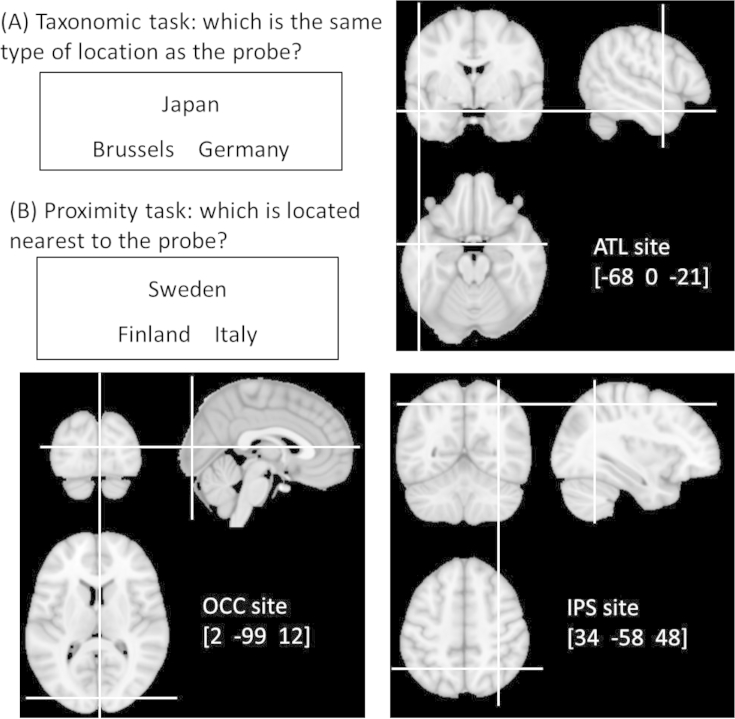
Illustration of experimental tasks and stimulation sites. Participants made taxonomic and proximity decisions to the names of cities, regions and countries. (A) Participants were asked to decide which of the two alternatives was the same type of location as the probe, irrespective of their location in the world. They were instructed to match cities with other cities and countries with other countries. (B) Participants were asked to decide which of the two alternatives was located closest to the probe.

Participants completed two matching tasks probing different aspects of geographical knowledge (see Fig. 1). Each task consisted of 50 Global and 30 UK trials. The taxonomic task required participants to select which of two alternatives was the same type of location as the probe. On Global trials, participants were instructed that they would be presented with cities and countries from around the world and they were to match cities with cities and countries with countries. On UK trials, they were presented with cities and regions within the UK and instructed to match cities with cities and regions with regions. The proximity task required participants to select which of two alternatives was located the shortest distance from the probe. On proximity trials, all locations were taken from the same taxonomic category (e.g., all were cities).

The tasks were designed as follows. We began by constructing 130 trials for each task (76 Global and 54 UK). Each trial featured the names of three locations: a probe and two options. The locations were cities and countries from around the world and cities and regions (predominately counties) from within the UK. For taxonomic trials, the target belonged to the same taxonomic category as the probe (i.e., country, city or region). The foil was from a similar geographical area as the target but belonged to a different category. Many UK counties have the suffix –shire (e.g., Lancashire). To prevent participants from matching locations using this suffix, we ensured that counties ending in –shire were paired with counties that did not use this suffix (e.g., Lancashire with Cornwall).

For proximity trials, the target was geographically close to the probe and the foil was distant. All three locations in the trial belonged to the same taxonomic category. For Global proximity judgements, there was a possibility that participants would use membership of larger geographical units (i.e., continents) to guide their decisions, rather than attending to the distances between locations. To prevent this, we ensured that the target and foil both came from the same continent.

These stimuli were piloted in 27 UK undergraduate students from the University of Manchester, which allowed us to eliminate trials with high error rates. The final set of stimuli, used in the TMS experiment, consisted of 80 taxonomic trials (50 Global; 30 UK) and 80 proximity trials (50 Global; 30 UK). The two tasks were matched for mean accuracy in the pilot study [*t*(158) = 1.15, *p* = .25; see Table 1 for means]. In addition, the mean length (number of letters) of the geographical terms used in each task was equivalent (*t* = .81, *p* = .42), as was their frequency of occurrence in the British National Corpus (*t* = .28, *p* = .78). We also used an online mapping tool to assess the geographical distance between probes and targets and probes and foils. For the proximity task, as intended, the mean distance from probe to target was shorter than the distance from probe to foil [Global trials: *t*(49) = 9.8, *p* < .001; UK trials: *t*(29) = 12.7, *p* < .001]. For the taxonomic task, there was no difference in the probe's distance from target and foil [Global trials: *t*(49) = .15, *p* = .89; UK trials: *t*(29) = 1.6, *p* = .12], indicating that spatial proximity did not act as a useful cue in this task.

### 2.3 Additional semantic and numerical knowledge tests

Although our main focus was on geographical concepts, we also probed knowledge for word meanings and numerical magnitudes, using tasks from previous TMS and fMRI studies (Binney et al., 2010, Hoffman et al., 2015, Hoffman et al., 2010, Pobric et al., 2007). In the word meaning task, participants were presented with a probe word and three options. They were asked to select the word that had a similar meaning to the probe (e.g., probe: recess; options: morsel, interval or assumption). In the numerical magnitude task, participants were presented with a probe number and three numerical options. They were asked to indicate which number was closest in magnitude to the probe. Each task consisted of 80 trials.

### 2.4 Design and procedure

Participants received stimulation to three sites, displayed in Fig. 1:

1. Left lateral anterior temporal cortex. Stimulation co-ordinates were taken from a study involving recognition of famous landmarks (Gorno-Tempini & Price, 2001). Stimulation to this area disrupts semantic processing for words and pictures (Pobric et al., 2007, Pobric et al., 2010a).
2. Right IPS. Co-ordinates were taken from a study in which participants estimated distances between features in a virtual environment (Mellet et al., 2010). Previous studies have reported disruption to numerical and spatial processing following stimulation to this region (Dormal et al., 2008; Gobel et al., 2001, Kadosh et al., 2007).
3. Occipital pole. This was included as a control site to assess non-specific effects of TMS, following previous TMS studies of semantic processing (Hoffman et al., 2012, Pobric et al., 2010a). This site corresponded to the Oz location in the International 10–20 EEG system.

Each site was stimulated in a separate experimental session, with the order counterbalanced across participants. In each session, participants first completed a short practice block of each of the four tasks, followed by a baseline assessment composed of 40 trials of each task. They then received 10 min of offline stimulation at 1 Hz, resulting in temporary disruption to cognitive functions supported by the stimulated region. During this window of disruption, they completed post-TMS assessments for each task.

All experimental tasks were administered on a PC running Eprime software. Each trial began with a blank screen presented for 500 msec, followed by a fixation cross presented for 250 msec. The probe was then presented on the screen with the two or three options in a line beneath it. Participants indicated their choice via button-press, with the response immediately triggering the next trial. If no response was made after 5250 msec, an error was recorded and the program continued onto the next trial. The experiment consisted of six blocks, each presented pre-TMS and post-TMS: Global proximity, UK proximity, Global taxonomic, UK taxonomic, word meaning, numerical. The order of the blocks was counter-balanced across individuals.

### 2.5 TMS protocol and stimulation parameters

Stimulation sites were localised in individual participants using a frameless stereotaxy system (Brainsight, Rogue Research Inc.). Landmarks on the participant's head were co-registered to their structural MRI scan using this system. Stimulation sites were defined using co-ordinates from previous neuroimaging studies (Gorno-Tempini and Price, 2001, Mellet et al., 2010). MNI co-ordinates for left anterior temporal cortex were [−68 0 −21]. Co-ordinates for right IPS were [34 −58 48]. The MNI co-ordinates were transformed into each participant's native brain space using SPM8. These co-ordinates were then used as stimulation targets and the TMS coil was placed on the corresponding location on the participant's scalp. Brainsight was used to track the position of the TMS coil throughout the stimulation period, ensuring that it remained on the target location.

The occipital pole control site was defined as the Oz location in the International 10–20 EEG system and was identified by finding the inion and then moving along the midline dorsally by 10% of the nasion-to-inion distance. For tracking purposes, this location was logged as a target using Brainsight and its location was later transformed into MNI space. The mean MNI co-ordinates for this site were [2 −99 12].

Stimulation was applied using a Magstim Rapid2 stimulator with a 70 mm figure-of-eight coil. Stimulation was applied at 65% of machine output for all participants, at 1 Hz for a total of 10 min.

### 2.6 Data analysis

Log-transformed reaction time data were analysed using linear mixed-effects models, after excluding errors (9% of responses) and any RTs falling more than two standard deviations outside a participants' conditional mean (4% of responses). Mixed-effects models simultaneously account for random effects across participants and items in a single model. Our analysis strategy followed the recommendations of Barr, Levy, Scheepers, and Tily (2013). We specified a maximal random effects structure for all models, including random intercepts for participants and items as well as random slopes for all factors that varied within-participant or within-item. The following control predictors were included in all models: trial position within block, block position within session, session order and accuracy on previous trial (as errors typically lead to a pronounced slowing on the subsequent trial). If a model failed to converge, random correlations were omitted from the model and it was re-estimated. The significance of particular effects was assessed by comparing the full model with a reduced model that was identical in every respect except for the exclusion of the effect of interest. If the fit of the full model was significantly better than that of the reduced model (assessed by a likelihood-ratio test) we considered the effect to be significant.

## 3 Results

### 3.1 Effects of TMS on geographical knowledge

**Fig. 2:**
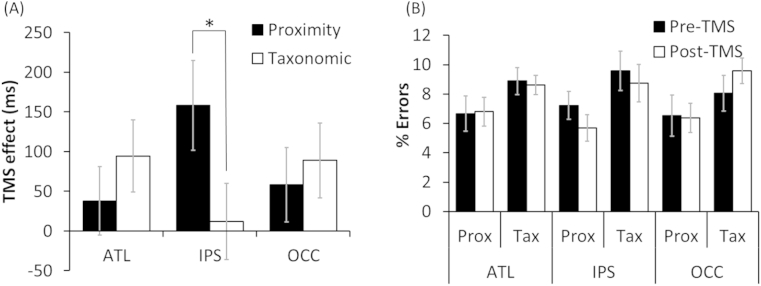
Effects of TMS on geographical decisions Bars indicate one standard error of the mean, adjusted to reflect the relevant variation in within-subject designs (Cousineau, 2005).

Fig. 2A shows the average TMS effect for each task following stimulation to ATL and IPS (effects for the OCC control site are discussed in the next section). Data were analysed in a 2 × 2 × 2 linear mixed-effects model that included task, stimulation site and TMS (before *vs* after) as factors. There was no effect of task (*χ*^2^ = .003, *p* = .96) indicating that the two tasks were similar in difficulty (mean for proximity = 1701 msec; taxonomic = 1697 msec). There was, however, a main effect of TMS (*χ*^2^ = 5.58, *p* = .018) and, importantly a significant three-way interaction (*χ*^2^ = 4.23, *p* = .040), indicating that the TMS effect varied with both site and task.

Follow-up tests investigated the effects at each site individually. ATL stimulation resulted in overall slowing in task performance (*χ*^2^ = 5.00, *p* = .024) but this effect did not interact with task (*χ*^2^ = .61, *p* = .44). When each task was considered individually, however, the TMS effect was only significant for the taxonomic task (taxonomic: *χ*^2^ = 5.14, *p* = .023; proximity: *χ*^2^ = 1.55, *p* = .21). Stimulation to IPS also had an overall slowing effect (*χ*^2^ = 3.87, *p* = .049), but in this case there was also a trend towards an interaction with task (*χ*^2^ = 2.89, *p* = .089). TMS had a significant effect on the proximity task (*χ*^2^ = 5.20, *p* = .023) but no effect on the taxonomic task (*χ*^2^ = .38, *p* = .54). To summarise, TMS to the ATL produced a slowing in processing of geographical concepts that did not differ between tasks, whereas TMS to the IPS had a highly selective effect on judgements based on geographical proximity.

### 3.2 Occipital control site

The occipital pole was stimulated as a control site for which we had no specific hypotheses. TMS effects for this site are shown in Fig. 2A. A 2 × 2 model was analysed, including task and TMS as factors. There was a main effect of TMS (*χ*^2^ = 8.26, *p* = .004), indicating that stimulation to this area slowed geographical decisions. There was no effect of task (*χ*^2^ = .03, *p* = .86) and no interaction (*χ*^2^ = .08, *p* = .78). As the effect of occipital TMS was unexpected, we investigated this effect in more detail. We found that there was a weak correlation between the size of the occipital TMS effect observed in individual participants (averaged across the two tasks) and participants' ratings of their own geographical knowledge (*r* = −.40, *p* = .09). In other words, participants who considered their geographical knowledge to be poor tended to show greater slowing when their occipital pole was stimulated. This suggests that recruitment of the occipital cortex may be a particular strategy employed by individuals with less developed geographical knowledge. No such correlations were found with the effects of ATL or IPS TMS (|*r|* < .05).

### 3.3 Errors

Error rates were below 10% in every condition (see Fig. 2B). Error rates were subjected to statistical analyses analogous to those performed on RTs but using ANOVA. There were no main effects of TMS or interactions between TMS and other factors. There was, however, a main effect of task [*F*(1,17) = 7.73, *p* = .013], as error rates were slightly higher for the taxonomic task.

### 3.4 Additional semantic and numerical knowledge tests

**Fig. 3:**
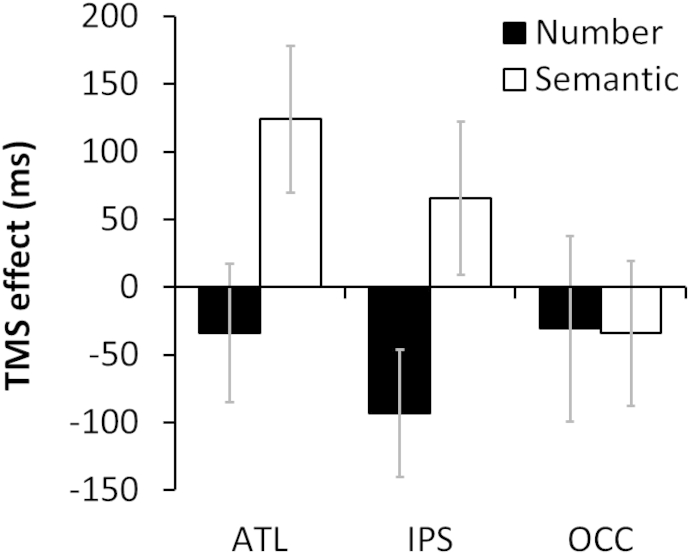
Effects of TMS on semantic and numerical judgements.

In each session, participants also completed supplementary tasks probing knowledge of non-geographical word meanings and numerical magnitudes. Mean RT for the word meaning task was 2053 msec and for the number task was 2114 msec. The effects of TMS on these tasks are shown in Fig. 3. RT data for the two sites of interest were analysed in a 2 (task) × 2 (site) × 2 (TMS) model. There were no main effects but there was a significant interaction between TMS and task (*χ*^2^ = 6.43, *p* = .011). Further analyses of the data for each site revealed that stimulation to the ATL showed a trend towards slowing word meaning judgements (*χ*^2^ = 3.35, *p* = .067) while there were no effects on number judgements (*χ*^2^ = .025, *p* = .87). This is consistent with previous findings (Lambon Ralph et al., 2009, Pobric et al., 2007, Pobric et al., 2009) and suggests that the ATL site stimulated in this study plays a functional role in verbal semantic processing.

Stimulation to the IPS had no effect on word meaning judgements (*χ*^2^ = 2.10, *p* = .15). In contrast, we found that IPS stimulation significantly *speeded* responses on the numerical judgement task (*χ*^2^ = 5.21, *p* = .022). TMS to this region in the left hemisphere is known to disrupt numerical cognition, though effects in the right hemisphere have been observed less consistently (Dormal et al., 2008; Gobel et al., 2001; Gobel, Rushworth, & Walsh, 2006). It is not clear why TMS had a facilitatory effect in this case. One possibility is that the particular numerical ability probed here is supported by left IPS rather than right IPS. As a consequence, inhibition of the function of right IPS may have allowed the left IPS to function more efficiently.

Finally, TMS to the occipital pole had no effect on either task (word meanings: *χ*^2^ = .01, *p* = .92; numbers: *χ*^2^ < .001, *p* = .99). This result stands in contrast to the significant effects of TMS to this region on geographical judgements.

## 4 Discussion

Geographical concepts are an important but poorly understood component of conceptual knowledge. We used rTMS to investigate the roles of ATL and IPS in processing this information. Both regions made a critical but distinct contributions to judgements about geographical locations. IPS demonstrated a selective involvement in processing the spatial relationships between locations but not in non-spatial aspects of their representation (e.g., their classification as a country or a city). In contrast, stimulation to the ATL produced general disruption to geographical concepts, which did not differ as a function of task. Unexpectedly, we also found that inferior occipital cortex played a role in processing these concepts for some individuals. These results have important implications for our understanding of the neural basis of geographical knowledge and its relationship with other forms of conceptual knowledge.

The ATL and IPS are major components in two distinct representational systems. While the ATL is critically involved in semantic knowledge for words and objects (Patterson, Nestor, & Rogers, 2007), IPS is involved in the representation of numerical and spatial magnitudes (Walsh, 2003). Typically, the functions of these two regions are highly dissociable under brain damage. Our results indicate, however, that geographical knowledge is supported jointly by the ATL semantic system *and* the parietal magnitude system. How do these two systems interact? One possibility is suggested by the “hub-and-spoke” model of conceptual knowledge (Patterson et al., 2007, Rogers et al., 2004). According to this theory, the different elements of experience that contribute to a particular concept are represented in primary association cortices distributed throughout the brain. Information about object shape is represented in ventral occipitotemporal regions, for example, and auditory characteristics in superior temporal cortex. These modality-specific regions are termed “spokes”. The ATL “hub” plays an important role in integrating these disparate sources of information into coherent concepts, permitting the extraction of supramodal conceptual relationships (Lambon Ralph, Sage, Jones, & Mayberry, 2010).

The selective involvement of IPS in spatial geographical judgements suggests that this region is acting as a previously unidentified “spoke” in the semantic system, coding information about the spatial relationships between locations. The ATL, on the other hand, appears to support representations of geographical concepts that are called upon in all geographical knowledge tasks, consistent with its role as a more general semantic “hub”. In fact, in numerical terms, the effect of ATL TMS was larger for the taxonomic task, suggesting that the ATL may place a less central role in location-based judgements. However, since the interaction of task and TMS was not significant for this site, we cannot be confident that there was a differential effect on the two tasks. This remains an important question for future research. In any case, on the view we have put forward, IPS and ATL interact to support judgements of geographical proximity. Similarly, other classes of concept that are strongly associated with particular types of experience selectively recruit other “spoke” regions. For example, anterior parietal and premotor regions associated with motor planning are selectively involved in knowledge for manipulable objects (Cattaneo et al., 2010, Pobric et al., 2010b) and regions of ventromedial prefrontal cortex involved in emotional processing are activated when people comprehend words with strong emotional valence (Vigliocco et al., 2014).

We also found that TMS to the occipital pole had a selective effect on geographical judgements. While this effect was unexpected, it suggests that other neural systems also contribute to geographical processing in some circumstances. Early visual cortex has been implicated in the generation of mental imagery (Kosslyn, Ganis, & Thompson, 2001). Specifically, TMS to the occipital pole has been shown to disrupt performance on visual imagery tasks (Kosslyn et al., 1999). It is possible, therefore, that participants in our study formed mental images of the locations involved (e.g., associated landmarks or scenes) to support their performance and that TMS to the occipital cortex disrupted this process. The correlation of the size of the occipital TMS effect with level of geographical knowledge in our participants further suggests that those with weak geographical knowledge were more heavily reliant on this strategy. Verification of this hypothesis requires further, more targeted, investigation, as it is not clear at present how imagery would support geographical processing and what types of mental image would be most beneficial to task performance. In some participants, imagery might take the form of a map of the spatial configuration of locations, for example, while others with weaker geographical knowledge might resort to imaging specific landmarks or scenes associated with the location being probed. It also seems plausible that visual imagery would benefit the proximity task to a greater degree, since this task involves processing of spatial relations. We found no evidence for this in the present study, but this remains an important hypothesis to explore in future work.

Finally, while we have focused on the roles of ATL and IPS in geographical concepts, this by no means rules out the involvement of other neural systems linked with scene processing and navigation. It is well known that the hippocampus and parahippocampal regions are involved in topographical learning and in the representation of scenes and environments (Burgess et al., 2002, Hafting et al., 2005, Maguire et al., 1998, O'Keefe and Nadel, 1978). The vast majority of studies have investigated knowledge acquired through direct navigation and exploration of real-word or virtual reality environments. They have also focused on topographical relationships on a local scale (e.g., navigation around a building, neighbourhood or town). It is entirely possible that the same systems are involved in supporting geographical knowledge on the more global scale investigated in the present study. However, there are two reasons why we would be wary of accepting this conclusion without empirical evidence. First, much of our knowledge for worldwide locations (and even cities in one's home country) is necessarily acquired indirectly, rather than by visiting these places in person. Second, even when direct experience is available, travel between locations hundreds of miles apart is a very different experience to travel within a local environment. A flight from, say, London to San Francisco does not provide the same rich set of topographical cues as a taxi ride from Buckingham Palace to the Tower of London. We also note that neuropsychological dissociations have occasionally been reported between verbal geographical knowledge and the ability to navigate in one's immediate environment. There are reports of patients who present with impairments of geographical knowledge despite intact ability to navigate in familiar and novel environments (Maguire & Cipolotti, 1998) and of the reverse pattern of deficits (dellaRocchetta et al., 1996, Habib and Sirigu, 1987). The potential role of medial temporal structures in supporting global geographical knowledge therefore remains an important open question for future investigation.

## Figures and Tables

**Table 1:** Mean (standard deviation) properties of trials in each condition.

	Taxonomic task		Proximity task	
Global trials	UK trials	Global trials	UK trials
Accuracy in pilot study	91% (6%)	87% (6%)	91% (5%)	89% (5%)
Word length (letters)	7.16 (1.40)	8.21 (1.3)	7.00 (1.1)	8.01 (1.4)
Word frequency (counts per million)	24.1 (29.1)	35.3 (33.5)	27.0 (24.1)	34.0 (33.4)
Probe-target distance (miles)	4580 (2574)	162 (101)	947 (1081)	56 (79)
Probe-foil distance (miles)	4560 (2486)	139 (68)	2880 (1899)	217 (68)
